# Antioxidant and Neuroprotective Properties of Non-Centrifugal Cane Sugar and Other Sugarcane Derivatives in an In Vitro Induced Parkinson’s Model

**DOI:** 10.3390/antiox10071040

**Published:** 2021-06-29

**Authors:** Javier Cifuentes, Vivian A. Salazar, Mónica Cuellar, María Claudia Castellanos, Jader Rodríguez, Juan C. Cruz, Carolina Muñoz-Camargo

**Affiliations:** 1 Department of Biomedical Engineering, School of Engineering, Universidad de Los Andes, Carrera 1 No. 18A-12, 111711 Bogotá, Colombia; jf.cifuentes10@uniandes.edu.co (J.C.); m.cuellar11@uniandes.edu.co (M.C.); mc.castellanos10@uniandes.edu.co (M.C.C.); 2 Department of Biochemistry and Molecular Biology, Faculty of Biosciences, Universitat Autònoma de Barcelona, 08193 Cerdanyola del Vallès, Spain; visalazar@uniandes.edu.co; 3 Department of Electrical and Electronics Engineering, School of Engineering, Universidad de Los Andes, Carrera 1 No. 18A-12, 111711 Bogotá, Colombia; 4 Corporación Colombiana de Investigación Agropecuaria—AGROSAVIA, Centro de Investigación Tibaitatá, km 14 vía Mosquera Bogotá, 250047 Mosquera, Colombia; jrodriguezc@agrosavia.co

**Keywords:** sugarcane, antioxidant, Parkinson’s disease, neuroprotective, mitochondrial membrane potential, non-centrifugal cane sugar

## Abstract

Non-centrifugal cane sugar (NCS) is a traditional sweetener in most sugarcane regions of the world. In Colombia, this product has a socio-economic importance due to the extensive cultivation area and the high consumption rate per capita. NCS traditional processing involves consecutive stages of thermal processing that begin with juice extraction, clarification, evaporation, and finish with syrup crystallization into a solid commercial product, identified as NCS. Sugarcane is known to have a natural content of polyphenols, amino acids, vitamins, minerals, and complex sugars, some of which are reported as antioxidant and antiproliferative agents thought to be responsible for the product’s bioactive profile. There is evidence to suggest that traditional thermal processing to obtain NCS leads to a considerable decrease in the contents of these bioactive compounds, mainly due to uncontrolled process variables such as temperature. Accordingly, the aim of this study was to assess and compare the bioactivity of sugarcane (SC) derivatives produced under controlled thermal conditions versus the traditional method. To achieve this goal, we evaluated the cytotoxic, antioxidant, and neuroprotective effects of varying concentrations of SC derivatives in an in vitro induced Parkinson’s model. Results demonstrate non-cytotoxic activity on the cellular model by 3-(4,5-dimethylthiazol-2-yl)-2,5-diphenyltetrazolium bromide (MTT) and LDH assays, even at the highest tested concentration of 8 mg/mL, for all SC derivatives. The effect of SC derivatives on the induced oxidative stress model showed a biological reversion and recovering effect of the mitochondrial membrane potential and a halting of the progress into the early apoptosis phase. In conclusion, we demonstrated that the bioactive compounds present in SC derivatives obtained by a process under controlled temperature conditions are largely preserved, and even their biological activities are enhanced compared with SC derivatives obtained by the traditional thermal evaporation of SC-juice.

## 1. Introduction

Sugarcane (*Saccharum officinarum* L.) is one of the most harvested crops in the world due to its nutritional value and versatility as a raw material for the food industry. This plant can be transformed into a variety of end-products to address a wide range of markets. It is harvested on approximately 26 million hectares distributed among more than 90 countries around the globe. The most common sugarcane-derived products or SC derivatives include cane sugar, juice, syrup, and non-centrifugal cane sugar (NCS). NCS is the technical term used by the Food and Agriculture Organization of the United Nations to refer to a non-refined, solid product minimally processed, obtained from the thermal evaporation of SC-juice [1,2]. Due to their sweetening properties, sugarcane (SC) derivatives have made it to the table of numerous consumers in North America and Europe. However, in the tropics and subtropics, the SC derivatives have represented an important part of traditional medicine for generations [3]. Nowadays, NCS is part of the essential diet of numerous countries in different latitudes around the globe, including the European Union, Thailand, Indonesia, Nigeria, Brazil, Peru, and Colombia. Especially for Colombia, NCS plays a main socioeconomic role, with an average consumption per person of 19 kg/year. Colombia is the largest NCS consumer per capita worldwide as well as the second largest producer worldwide after India, with 1.098 millions of tons/year [4,5,6,7]. 

NCS products contain several minerals, vitamins, organic acids, and amino acids, and their consumption has been proven beneficial for human health [5]. In this regard, recent studies have demonstrated antioxidant activity, which has been mainly attributed to the presence of polyphenols and flavonoids [8,9,10,11]. Moreover, a few reports describe the possible role of the Iron-containing minerals (Fe) in the production of new red blood cells [2]. Furthermore, several studies have reported cytoprotective, anti-carcinogenic, and immunomodulatory properties [2,3,12]. Despite the potential benefits of NCS products and due to their thermolability, the functionality of possible pharmacological components is largely compromised as processing generally proceeds at high temperatures [8,13,14]. In addition, the high variability on the content of bioactive molecules in NCS products, associated with traditional production processes with uncontrolled conditions, impedes the production of high quality and consistent products. To overcome this major hurdle, several innovative processing strategies have been developed, including the design of open pans with different geometries, multiple effect evaporators, and improved burner technologies that allow high energetic efficiency and accurate control of thermal profiles [15]. Over the past five years, we have been working on the development of technical strategies that significantly improve the traditional production processes where we designed (with the aid of mathematical models and computational simulations) and implemented technologies, such as the Ward-Cimpa chamber, that exhibit high energetic performance under controlled operation conditions [15,16]. With this approach, we were able to produce the NCS products at 94.5 ± 3.24 °C of experimental efficiency of combustion, with low energy lost, thereby resulting in a strategy that generates a significant decrease in the production costs. Moreover, through this process, it is possible to avoid degradation processes and, consequently, produce a high quality and consistent products [17].

Regarding the bioactive compounds of SC derivatives, polyphenols and flavonoids are the most representative in terms of pharmacological activity. These polyaromatic compounds are capable of stabilizing reactive oxygen species (ROS), which can cause intracellular damage or even cell death. In particular, ROS can cause mitochondrial dysfunction and oxidation of several macromolecules within cells. This phenomenon has been previously associated with the progression of different conditions such as Parkinson’s disease (PD), Alzheimer´s disease (AD), and cancer. According to this, the reported antioxidant activity of SC derivatives can be explored on neuroprotective applications as they can be potentially useful to delay the neurodegenerative processes of Parkinson’s disease. In this disorder, neurons located in the substantia nigra of the brain, predominantly dopamine-producing neurons, are affected by three classical dysfunctions: oxidative stress, mitochondrial respiration defect, and abnormal protein aggregation [18]. The patient’s symptomatology encloses involuntary movements, slowness, and balance problems that are conventionally treated with levodopa (L-DOPA), whose continued use is related to increased tolerance and other side effects. Mimicking the degeneration of dopaminergic neurons in an in vitro PD model is critical for the development and assessment of novel bioactive compounds for the prevention and treatment of this pathology. The closer the resemblance of the in vitro PD model with the physiopathology observed in vivo, the higher the chances for clinical translation. In this regard, several substances have been used to induce or simulate the behavior of PD for most accurate approaches at the in vitro and in vivo levels. Rotenone, a compound generally derived from plants from the genuses *Lonchocarpus*, *Derris*, and *Tephrosia*, is particularly one of the most popular ones to cause oxidative stress and eventually apoptosis [19]. 

This study was therefore dedicated to identify and quantify phenolic compounds, along with the evaluation of the antioxidant and neuroprotective potential of SC derivatives (SC juice, syrup and NCS), processed under controlled temperature conditions (constant heat flux). Toward this end, we used SH-SY5Y neuroblastoma cells with rotenone-induced exacerbated oxidative stress as an in vitro PD model [20,21]. This line has been broadly used for this purpose due to their close resemblance to dopaminergic neurons [19]. In addition, we conducted enzymatic activity studies for the isoform B of the monoaminoxidase enzyme (MAO-B), which is involved in the metabolism of serotonin and dopamine. MAO-B is located in the outer mitochondrial membrane and has been reported to malfunction as PD progresses [22,23]. To our knowledge, this study intends to validate, for the first time, SC derivatives as highly-accessible therapeutic agents for the delay of neurodegeneration in Parkinson’s Disease.

## 2. Materials and Methods

### 2.1. Materials

SH-SY5Y neuroblastoma (CRL-2266^®^, Vernon, CA, USA) cells were obtained from ATCC (Manasas, Virginia). Dulbecco’s modified eagle’s medium (DMEM), fetal bovine serum (FBS), and trypsin EDTA were obtained from Biowest (Riverside, MO, USA). Penicillin/Streptomycin (P/S) was purchased from Lonza. DPPH free radical, JC-1 iodide dye, and Trolox were obtained from Santa Cruz biotechnology (Santa Cruz, CA, USA). Dimethyl sulfoxide (DMSO, 99.9%), Thiazolyl Blue Tetrazolium Bromide (MTT, 98%), and Rotenone were purchased from Sigma–Aldrich (St. Louis, MO, USA). Propidium iodide and Hoechst 33,342 Staining Dye Solution were purchased from Invitrogen (Carlsbad, CA, USA). LDH (Lactate dehydrogenase) and Fluoro: MAO (Monoamine oxidase) kits were obtained from Roche and Cell technology (Hayward, CA, USA), respectively. Reference standards for analytical HPLC-MS: xanthines: caffeine (Part No. C8960-250G, Sigma–Aldrich), theobromine (Part No. T4500-25G, Sigma–Aldrich), and theophylline (Part No. T1633-25G, Sigma–Aldrich); catechins: (±)-catechin (C) (Part No. C1788-500MG, Sigma–Aldrich), (−)-pigallocatechin gallate (EGCG) (Part No. E4143-50MG, Sigma–Aldrich), (−)-epicatechin (EC) (Part No. E1753-1G, Sigma–Aldrich), (−)-epicatechin gallate (ECG) (Part No. E3893-10MG, Sigma–Aldrich), (−)-epigallocatechin (EGC) (Part N E3768-5MG, Sigma–Aldrich); flavonoids: caffeic acid (Part No. C0625, Sigma–Aldrich), p-coumaric acid (Part No. C9008, Sigma–Aldrich), rosmarinic acid (Part No. 536954-5G, Sigma–Aldrich), quercetin (Part N Q4951-10G, Sigma–Aldrich), naringenin (Part No. N5893-1G, Sigma–Aldrich), luteolin (Part No. L9283-10MG, Sigma–Aldrich), kaempferol (Part No. K0133-50MG, Sigma–Aldrich), pinocembrin (Part No. P5239, Sigma–Aldrich), apigenin (Part No. A3145-25MG, Sigma–Aldrich); and anthocyanins: 3-rutinoside cyanidin (Part No. G36428, Sigma–Aldrich), pelargonidin 3-glucoside (Part No. 53489, Sigma–Aldrich), and quercetin 3-glucoside (Part No. 89230, Phytolab).

### 2.2. Sugarcane-Derivatives from Controlled Laboratory Conditions

**Scheme 1:**
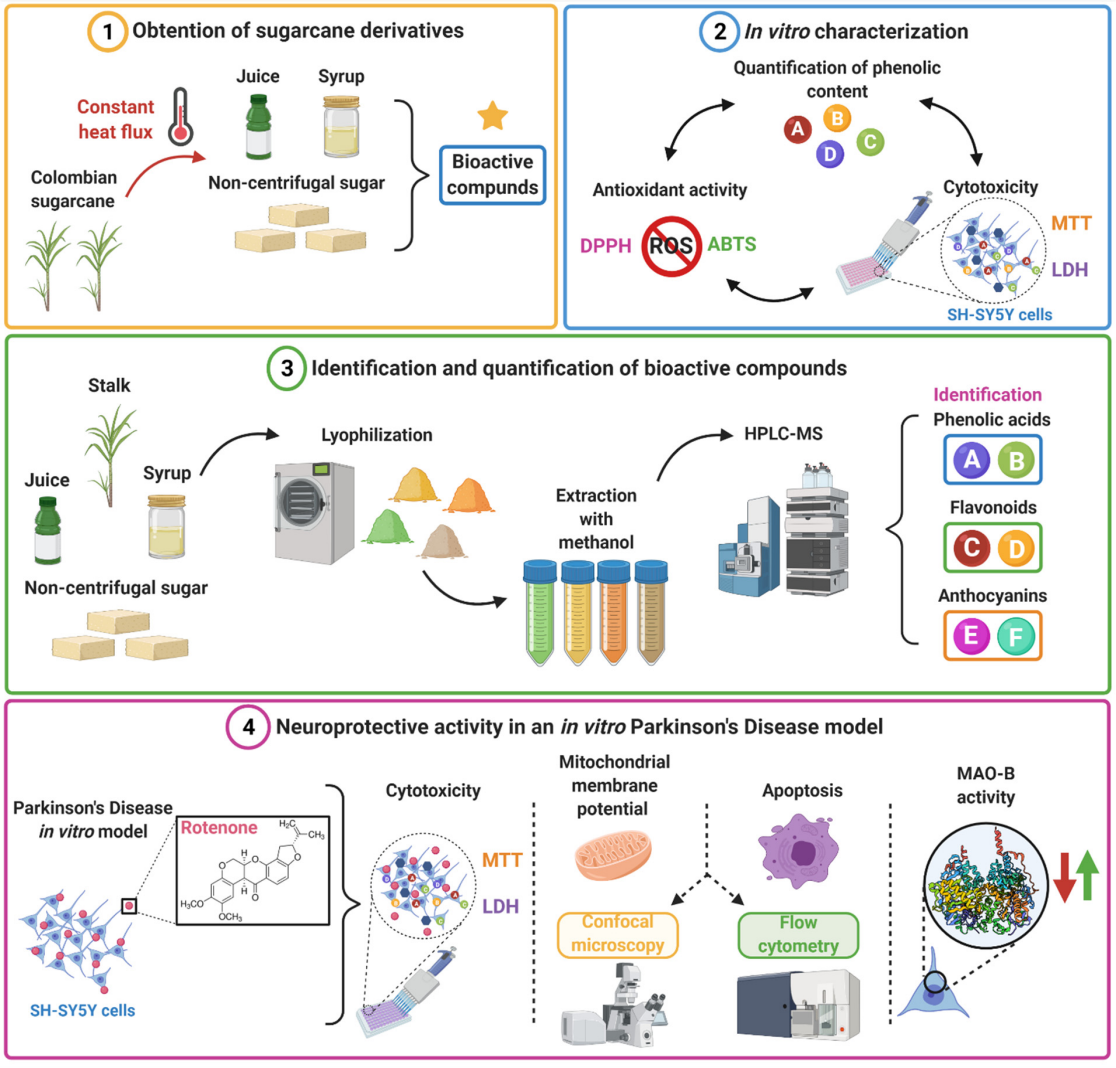
Illustration of the developed workflow for validating SC derivatives as highly-accessible therapeutic agents for the delay of neurodegeneration in PD (Created with Biorender.com, accessed 1 June 2021). (1) The sugarcane is processed by a thermally controlled process to obtain juice, syrup, and NCS containing the bioactive compounds. (2) A preliminary characterization of biological response is conducted in vitro that included assays for antioxidant activity, phenolic content, and cytotoxicity. (3) Bioactive compounds were extracted by methanolic extraction and, subsequently, analytical HPLC-MS were carried out for identification and quantification. (4) The neuroprotective activity of SC derivatives was evaluated in vitro in a rotenone-induced model via mitochondrial membrane potential, apoptosis, and MAO-B activity.

Sugar cane (SC) derivatives were obtained after the clarification (Juice at 21 °Brix), evaporation (syrup at 68 °Brix), and concentration (NCS with 93 °Brix) stages in the NCS processing using controlled laboratory conditions (LC) of the traditional process (Scheme 1). Samples were obtained from three subsequent NCS production batches in order to have three independent replicates for analysis. Prior to use, each sample was subjected to physicochemical, organoleptical (i.e., moisture content, density, dissolution capacity, impurities content, texture, and color), and microbiological (counting mesophilic aerobes, fecal and total coliforms, fungi, and yeasts) evaluations.

### 2.3. SC Derivatives Sample Preparation

All SC derivatives samples were lyophilized in a FreeZone 12 L −84 °C Console Freeze Dryer with Stoppering Tray Dryer (Catalog # 711221010) until the water content was below 10% (results not shown). Sugarcane stalk was pulverized in a 6875 Freezer/Mill^®^ High-Capacity Cryogenic Grinder (Metuchen, NJ, USA). Samples were subsequently stored at −80 °C until further use. Prior to use in cell culture assays, 10 mg of each SC derivative was resuspended in Dulbecco’s Modified Eagle Medium (DMEM), sterilized using a 0.22 μm filter, and used as stocks for further serial dilutions.

### 2.4. Quantification of Phenolic Content via the Folin–Ciocalteu Method

The total phenolic content of each sample was determined according to the Folin–Ciocalteu method [24]. Briefly, 1 mL of sample (final concentration between 5 to 10 mg/mL) was added to 6 mL of distilled water followed by the addition of 500 µL of a 1:10 dilution of Folin–Ciocalteu reagent. The reaction mixture was pre-incubated for 3 min at room temperature, and then 1 mL of 20% (*w*/*v*) Na_2_CO_3_ was added and mixed by inversion. After 90 min of reaction in the dark, the absorbance was measured at 725 nm. The total phenolic content was determined according to a caffeic acid standard curve. This procedure was repeated for each sample.

### 2.5. Identification and Quantification of Phenolic Compounds by Analytical HPLC-MS

#### 2.5.1. Preparation of Phenolic Extracts

The extraction of phenolics compounds was performed with methanol as the organic solvent extractant (Scheme 1). Samples were first lyophilized to complete drying. Then, 30 g of each dried sample were suspended in methanol (1:1 *w*/*v*) and subsequently homogenized at 10,000 rpm for 1 min in an ice water bath. Next, extracts were filtered under reduced pressure through filter paper (Whatman No. 1) to remove solid contaminants. In addition, extracts were concentrated by removing excess of solvent using a rotatory evaporator at 37 °C and 100 rpm for 30 min. Finally, concentrated extracts were diluted in 60 mL of methanol and stored at 4 °C until further use.

#### 2.5.2. Analytical Liquid Chromatography-Mass Spectroscopy

The preparation of the samples was carried out by dissolving the extracts in a solution consisting of methanol: water (0.2%) in formic acid (1:1), followed by vortex for 5 min and ultrasonication for 5 min. The analysis was performed on a Dionex Ultimate 3000 UHPLC system (Thermo Scientific, Sunnyvale, CA, USA) equipped with a binary bump (HP G3400RS), a temperature-controlled sampler/auto injector (WPS 300TRS), and a temperature regulated column compartment maintained at 30 °C (TCC 3000) coupled with an Obitrap mass spectrometer (Exactive Plus, Thermo Scientific, Sunnyvale, CA, USA). MS analysis was carried out using an electrospray ionization interface (ESI) in positive ion mode with a voltage of 4.5 kV. Separation was carried out on a Hypersil GOLD Aq column (Thermo Scientific, Sunnyvale, CA, USA; 100 mm × 2.1 mm, 1.9 μm). Mobile phases used were A: aqueous solution containing 0.2% formic acid and B: acetonitrile with 0.2% ammonium formate with the following gradient program: 100% A (4 min), changing linearly to 100% B (8 min), returning to the starting conditions in 1 min, a complete running time of 13 min with 3 min for post-running. Nitrogen was used as the sheath and auxiliary gas. Mass spectra were recorded with a resolution of 30,000 in full scan mode in a mass range from *m*/*z* 60 to 900. Identification of the phenolic compounds was determined by comparison of retention time and mass measurements (Δppm < 0.001) with reference standards and extracted ion technique (EIC). Results were presented as concentration of compounds (mg/kg) with the corresponding error propagation analysis as estimated from the instruments precision (%).

### 2.6. SC Derivatives Free Radical Scavenging Antioxidant Activity Assessment

#### 2.6.1. DPPH Assay

To measure the radical-scavenging capacity of each sample (Juice, syrup, and NCS), the spectrophotometric DPPH (2,2-diphenyl-1-picrylhydrazyl) method was used (Scheme 1) [10,25]. Each sample was dissolved to an initial concentration of 8 mg/mL in methanol and then serially diluted 1:2 down to 7.8 µg/mL. The resulting samples were mixed 1:1 with 0.2 mM methanolic DPPH solution. The plate was incubated for 30 min in the dark at ambient temperature, and the absorbance was then recorded at 517 nm using a Synergy HT Multi-Mode Reader (BioteK, Winooski, VT, USA) spectrofluorometer. An ascorbic acid standard curve was built for concentrations ranging from 7.81 µg/mL to 1000 µg/mL.

The DPPH radical scavenging activity (% inhibition) was calculated as follows:

(%) inhibition = (1 − [(Ac − As)/Ac]) × 100(1)

where Ac is the absorbance of control (DPPH + Methanol without sample), and As is the absorbance of sample (DPPH + Sample (extract/standard)). In order to assess the antiradical potency, the IC_50_ was determined, which was defined as the concentration of substrate that causes 50% reduction of the DPPH color absorbance.

#### 2.6.2. ABTS Assay

A 7 mM 2,2′-azino-bis (3-ethylbenzothiazoline-6-sulfonic acid) (ABTS) stock solution was prepared in distilled and deionized water (Scheme 1). ABTS•+ was produced by mixing the ABTS stock solution with 2.45 mM potassium persulfate (final concentration) and allowing the mixture to react in the dark at room temperature for 12–16 h before use [26]. Next, the ABTS•+ working solution was diluted with phosphate-buffered saline 5 mM, pH 7.4 to obtain an absorbance of 0.70 OD at 734 nm. Subsequently, 180 μL of diluted ABTS•+ was added to 20 μL of the sample. Absorbance was read after five minutes of incubation at room temperature. Trolox was used as positive control. This activity was measured as percentage (%) inhibition (ABTS scavenging), which was calculated as follows:

% inhibition = [1 − (Asample − Acontrol)/Acontrol] × 100(2)

### 2.7. Cell Culture and Cytotoxicity of SC Derivatives

The SH-SY5Y neuroblastoma cell line was cultured and maintained in 75 cm^2^ flasks with DMEM media supplemented with FBS 10% at 37 °C, in a humidified atmosphere with 5% CO_2_ and 95% air, until the cells reached a confluence of 90–95%.

### 2.8. MTT Assay

Cell viability in the presence of SC derivatives was determined by mitochondrial metabolic activity, as evaluated by the 3-[4,5-dimethylthiazole-2-yl]-2,5-diphenyltetrazolium bromide (MTT) assay (Scheme 1). SH-SY5Y cells were seeded at a density of 1 × 10^4^ cells per well on a 96-well microplate with DMEM supplemented with 10% SBF and subsequently incubated for 24 h to allow cell attachment. Cells were subsequently treated with SC derivatives samples dilution ranging from 0.5 to 6.5 mg/mL in the absence of FBS and incubated over 24 and 48 h. DMEM and 10% DMSO in DMEM were used as negative and positive controls, respectively. Once the incubation period was completed, 10 µL of 0.5 mg/mL MTT in PBS were added to each well and incubated for 3 more hours. Supernatants were removed and any MTT crystals formed were dissolved with 100 µL 100% DMSO. Absorbance was measured at 595 nm in a Thermo Scientific Multiskan™ FC Microplate spectrophotometer. Cell viability percentage was calculated as follows:

% Viability = (Absorbance (treated cells)/Absorbance (negative control)) × 100(3)

### 2.9. LDH Assay

Cell viability, as determined by cell membrane integrity, was assessed by quantifying lactate dehydrogenase (LDH) activity using a commercially available kit (Scheme 1). The manufacturer’s recommendations were followed with minor modifications. Briefly, SH-SY5Y cells were treated with 1:2 serial concentrations of SC derivatives samples (from 0.5 to 6.5 mg/mL) and incubated for 24 and 48 h. Once incubation time was reached, 50 µL of each cell supernatant was transferred to a new plate, mixed with 50 µL of reagent, and incubated in the dark for 20 min at room temperature. The absorbance from the released LDH was quantified at 490 nm wavelength and 650 nm as reference wavelength with the aid of a microplate reader (Thermo Scientific Multiskan™ FC Microplate spectrophotometer). Cells without treatment (DMEM) were the negative control while cells treated with 0.1% Triton X-100 were the positive one.

Percentage cell viability was calculated as follows:

% Viability = (1 − (Absorbance (treated cells)/Absorbance (positive control))) × 100(4)

### 2.10. Neuroprotective Effects in SH-SY5Y Cell Parkinson’s Disease Model (PD-Induced Model)

To assess the neuroprotective effects of SC derivatives, an in vitro PD-induced model was established in SH-SY5Y exposed to rotenone prior to treatment with SC derivatives (Scheme 1). This cell line has been broadly used to mimic PD in vitro due to its close resemblance to dopaminergic neurons and the possibility of inducing an oxidative stress similar to that of neurodegenerative processes caused by the disease [20,21]. Unless otherwise specified, rotenone-induced oxidative stress was achieved by exposing 100 µL of 200,000 cells/mL previously seeded on a 96-well microplate to 150 μM of rotenone for 24 h, as reported elsewhere for models of PD on SH-SY5Y cells [27]. The neuroprotective effect of SC derivatives was assessed by comparing changes in cytotoxicity, apoptosis, and membrane potential, and MAO-B activity in the presence of varying concentrations of the SC derivatives for rotenone-treated and healthy cells. For all assays, healthy cells were used as negative controls and cells with rotenone-induced PD were used as positive controls.

#### 2.10.1. PD Model Cell Viability after Exposure to SC Derivatives

To determine the capacity of SC derivatives to protect SH-SY5Y neuroblastoma cells against rotenone-induced cytotoxicity (PD-induced model), cell viability was evaluated in the presence of varying concentrations (31.25 to 1000 μg/mL) of SC derivatives and quantified by MTT and LDH assays, as described above.

#### 2.10.2. Apoptosis

The apoptotic effect of rotenone as well as the protective effect of SC derivatives was determined via confocal microscopy (Olympus FV1000 confocal laser scanning microscope (CLSM)) and flow cytometry (FACS CANTO BD) (Scheme 1). The cells were seeded, and the SC derivatives treatments added under the same conditions mentioned for cell culture and cytotoxicity assays above. Apoptosis by confocal microscopy using Hoestch dye was assessed by morphological changes in the cells nuclei. The area and perimeter of the selected cell nuclei was evaluated with the aid of the Fiji-ImageJ^®^ [28].

#### 2.10.3. Apoptosis by Flow Cytometry

In order to detect late apoptosis, the cells were stained using propidium iodide according to the method described by Bots et al., with some modifications (Scheme 1) [29]. The PD-induced model was treated with 1 mg/mL of the SC derivatives. Cells were washed once with ice-cold PBS (4 °C, pH 7.4) and collected by centrifugation at 250× *g* for 5 min (~1.2 × 10^6^). The pellet was then treated with the Nicoletti buffer (Na_3_C_6_H_5_O_7_, Propidium iodide and 10% (*v*/*v*) Triton X-100). Cells were subsequently kept in the dark at 4 °C for 1 h. Samples were acquired at low speed, and 20,000–30,000 events were collected in the same flow cytometer.

#### 2.10.4. Membrane Potential by Fluorescence Quantification and Confocal Microscopy

The PD-induced model was treated with SC derivatives in a broad range of concentrations (31.25–1000 µg/mL). Fluorescence was read at excitation/emission wavelengths of 485/530 nm and at 530/590 nm in MicroMax 384 Microwell-Plate Reader. Ratios of the fluorescent response 485/530 and 530/590 were then determined (Scheme 1).

For confocal microscopy, 50,000 cells per well were seeded on a glass slide, transferred to a 24-well microplate, and subsequently incubated for 24 h to allow cell adhesion. PD-induced model cells were exposed to the SC derivatives treatments (1 mg/mL) in DMEM for an additional 24 h. Subsequently, treated cells were washed three times with DMEM and exposed to DMEM with Hoechst (1:1000) and JC-1 dye (2 µM) for 30 min prior to observation under the confocal microscope. Untreated cells were used as negative control, and cells exposed to rotenone alone were used as positive control. The images were obtained with a 40X objective (NA = 0.6). Excitation/Emission wavelengths of 358/461 nm, 550/600 nm and 485/535 nm were used for the detection of nuclei, non-apoptotic cells and apoptotic cells, respectively. Three replicas were made for each treatment, and 10 images for each replica (10 cells per image) were taken. The ratio between red and green fluorescence was then calculated.

#### 2.10.5. Membrane Potential by Flow Cytometry

The PD-induced model was treated individually with the different SC derivatives at a concentration of 1 mg/mL. After treatment, cells were trypsinized, incubated with JC-1 as described above, and subsequently washed with PBS. A total of 25,000 events were acquired and analyzed in the same flow cytometer (Scheme 1) [29].

#### 2.10.6. MAO-B Activity Assay

PD-induced model cells were treated with serial dilutions of the SC derivatives (65.2–8000 µg/mL) and control samples (no treatment) and incubated for an additional 24 h (Scheme 1). Cells were then lysed by the frost-defrost method followed by differential centrifugation to isolate samples containing the MAO-B enzyme from cellular debris. The collected extracts were subsequently transferred to a new container for storage at −80 °C until further use.

The functional MAO-B activity in 25 µL samples of the cell lysates (around 0.4 mg/mL of total protein) was determined using the Fluoro:MAO^TM^ kit from Cell Technology, following the manufacturer’s recommendations, with minor modifications. The substrate for the enzymatic reaction was Tyramine. The MAO-B inhibitor pargyline was used as positive control, while the reaction sample buffer was the negative one. The product formed was determined from a hydrogen peroxide (H_2_O_2_) calibration curve. One unit of MAO-B catalyzes the formation of 1 µmole of (H_2_O_2_) per minute under assay conditions (Unit/mL = µmol/min/mL). MAO-B activity is presented relative to the negative control, which corresponds to 0% inhibition (i.e., 100% MAO-B activity).

### 2.11. Statistical Analysis

All data measurements are reported as mean ± standard deviation unless stated otherwise. Data analysis was performed using the Graph Pad Prism V 6.01 software (GraphPad Software, La Jolla, CA, USA). Statistical comparisons were made using ANOVA followed by post treatment (according to data, Dunn’s Multiple Comparison test or Tukey). Results with a *p*-value ≤ 0.05 (*) were considered significant.

## 3. Results

### 3.1. Polyphenolic Content and Free Radical Scavenging Properties SC Derivatives

The content of readily oxidizable compounds present in SC, SC juice, SC syrup, and Agrosavia NCS samples, as quantified by the Folin–Ciocalteu assay, are shown in Table 1. Additionally, Table 1 shows the free radical scavenging activity of each SC derivative as determined by DPPH and ABTS assays. For both assays, the antioxidant activity is reported as the (IC_50_) concentration, which gives half-maximal response. According to our results, the highest free radical scavenging activity was presented by SC juice, followed by SC syrup and Agrosavia NCS. The results are in accordance with expectations, most likely due to the mild temperature processing conditions used to obtain these derivatives [8,21]. No correlation was found between the total content of oxidizable compounds (polyphenols) and the antioxidant activity, as can be seen in Table 1. This result can be explained because the Folin–Ciocalteu assay is not specific for polyphenols and instead it also measures non-phenolic compounds that are readily oxidizable, such as 2-furanmethanol, furfural, or 5-(hydroxymethyl) furfural [24].

### 3.2. Identification and Quantification of Phenolic Compounds by Analytical HPLC-MS

HPLC-MS was carried out to identify the polyphenolic compounds present in the SC derivatives extracts (SC Juice, SC syrup, SC Agrosavia). Table 2 shows 12 different phytochemical molecules (phenolic acids, flavonoids, and anthocyanins) identified and quantified in all the SC derivatives. This analysis allowed us to identify 5 phenolic acids (p-hydroxybenzoic acid, vanillic acid, p-coumaric acid, feluric acid, and rosmarinic acid), 4 flavonoids, (luteolin, kaempferol, naringenin, and apigenin), and 3 anthocyanins (3-rutinoside cyanidin, cyanidin, and pelargonidine).

Raw SC stalk, compared to the SC derivatives, presented the highest concentrations of vanillic acid (84.2 mg/kg), p-coumaric acid (8.2 mg/kg), kaempferol (1.1 mg/kg), and naringenin (0.17 mg/kg). Meanwhile, SC syrup showed the highest concentrations of feluric acid (2.7 mg/kg) and pelargonidine (0.43 mg/kg). The obtained results show a significant decrease in content of phenolic acids such as p-hydroxybenzoic acid, feluric acid, luteolin, cyanidin, and pelargonidine after comparing the SC stalk with the different SC derivatives. This is most likely due to the technique employed to obtain the juice and to the process of clarification, in which several bioactive molecules present in the bagasse and in several antioxidant rich-waxes are discarded. Additionally, some important molecules with high antioxidant and neuroprotective potential, such as feluric acid and p-hydroxybenzoic acid, were present in SC syrup at higher concentrations than the others SC derivatives. We hypothesized that this phenomenon can be explained by the water evaporation process that takes place during syrup production, which leads to an increase in the concentration of phenolic compounds. In addition, the significant concentration decrease of most compounds initially present in Agrosavia NCS after thermal processing supports the hypothesis that the high temperatures used in this step compromised the stability of bioactive compounds.

### 3.3. Cytotoxicity of SC Derivatives

**Figure 1:**
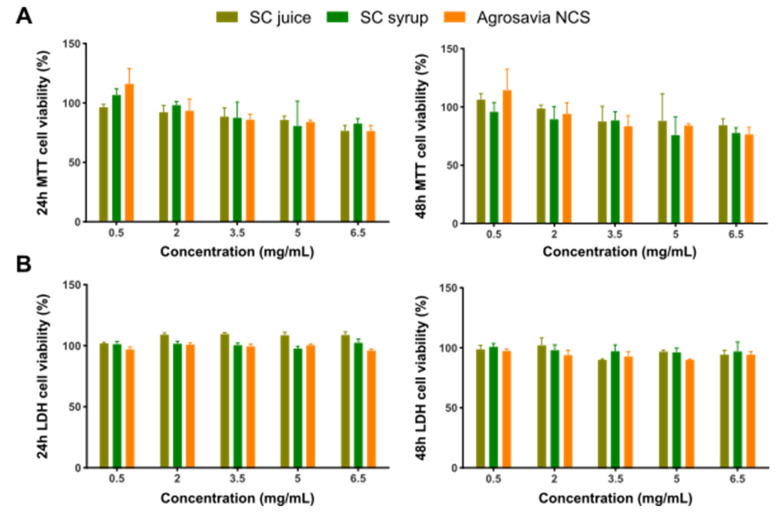
Neuroblastoma (SH-SY5Y) cell viability after exposure to Agrosavia SC derivatives, as measured by MTT (**A**) and LDH (**B**) assays. Cells were incubated with different concentrations of the samples for 24 and 48 h. Results are presented as mean ± SEM of 3 independent experiments. Two-way ANOVA test was used to assess statistical significance (*p*-value ≤ 0.05).

The potential cytotoxic effect of the SC derivatives (SC stalk, SC juice, SC syrup, and Agrosavia NCS) was determined via thiazolyl blue tetrazolium bromide (MTT) and lactate dehydrogenase (LDH) assays. These two techniques allowed us to estimate mitochondrial function and cell membrane integrity, respectively. As shown in Figure 1, none of the evaluated concentrations showed cytotoxic activity after 24 h of exposure by either of the two approaches evaluated. Similarly, after 48 h of exposure, the same trend is observed for the MTT and LDH assay results of all sample concentrations. Statistical analysis showed that there is no statistically significant difference between the treatments.

### 3.4. Recovery Effect of a Rotenone PD-Induced Model Exposed to SC Derivatives

**Figure 2:**
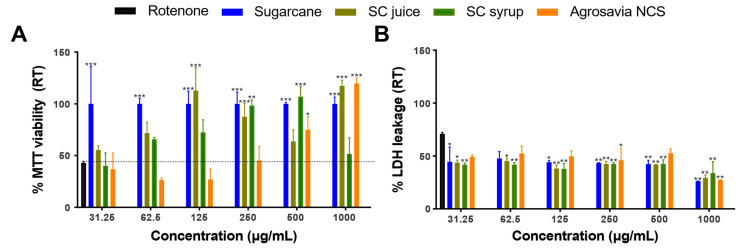
Recovery effect in a rotenone PD-induced model (RT) exposed to SC derivatives. (**A**) Cell viability as determined via MTT. The rotenone effect is highlighted by the horizontal black dotted line. (**B**) Cell membrane integrity determined via LDH released. The percentages were calculated as described in Material and Methods. The LDH released for the rotenone-induced control was 70 ± 0.79%. Results are presented as mean ± SEM of three independent experiments. Two-way ANOVA test was used to assess statistical significances * (*p*-value ≤ 0.05), ** (*p*-value ≤ 0.01) and *** (*p*-value ≤ 0.001).

Based on the innocuous effect of the SC derivatives on cell viability, the next step was to evaluate their neuroprotective effectiveness on the PD-induced model (via rotenone) using neuroblastoma cells. We established the optimum experimental conditions of rotenone injury at 150 μM (IC_50_) of rotenone dover 24 h. According to the MTT results (Figure 2A), treatment with rotenone led to about 60% reduction in viability. At concentrations above 250 μg/mL, SC derivatives (Agrosavia NCS, SC juice, SC syrup, and SC stalk) induced a viability recovery (63.6% to 106.8% of viability at 500 μg/mL) of injured cells. Except for SC syrup, a maximum in recovery was achieved at a concentration of 1000 μg/mL. Importantly, SC derivatives show recovery at all evaluated concentrations. This finding was also confirmed by LDH assays where maximal recovery was observed at a concentration of 1000 μg/mL for all derivatives (i.e., a release of LDH of only about 30% on average compared with 70% for the rotenone control). At such concentration, SC derivatives and Agrosavia NCS appear to have the highest effect. For all the other concentrations, the LDH release approached 45% on average with a slight increase (to about 50%) for Agrosavia NCS at 250 and 500 μg/mL.

### 3.5. Mitochondrial Membrane Potential Recovery on the PD-Induced Model Treated with SC Derivatives

**Figure 3:**
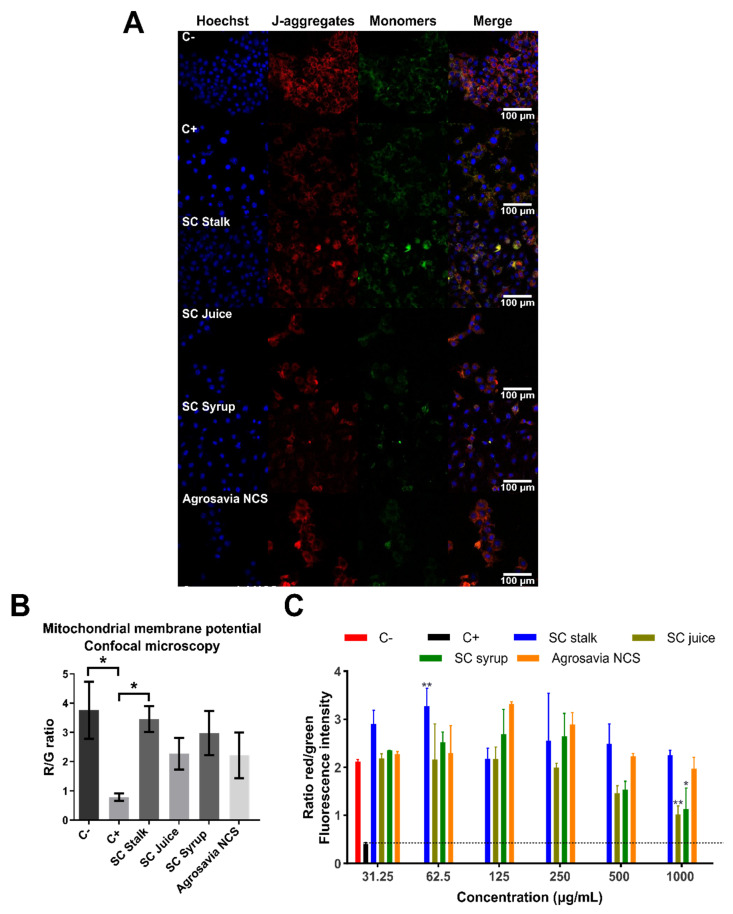
Changes in mitochondrial membrane potential. Negative control (C-): cells without rotenone treatment. Positive control (C+): rotenone. (**A**) Neuroblastoma (SH-SY5Y) cells were treated with rotenone (150 μM for 24 h). Posteriorly, the cells were washed with PBS 1X and incubated with the SC derivatives (1 mg/mL for 24 h). (**B**) R/G (Red fluorescence/Green fluorescence). Control (untreated cells) emitted red mitochondrial fluorescence with a small amount of green fluorescence (generated by a decrease in mitochondrial membrane potential) 3.759 ± 0.977 ratio. Cells treated with SC derivatives, where mitochondrial depolarization is generated, but the red fluorescence is still greater than the green one (3.455 ± 0.442 for SC stalk, 2.267 ± 0.537 for SC juice, 2.979 ± 0.758 for SC syrup, and 2.219 ± 0.784 for Agrosavia NCS). A significant decrease in the R/G ratio of the rotenone treatment (positive control) is observed compared to the negative control (healthy cells) and the SC stalk treatment. (**C**) Fluorescence intensity ratio of aggregates/monomers (red/green) in injured cells (SH-SY5Y neuroblastoma cells treated with rotenone) treated with SC derivatives as a function of concentration. Red and black columns represent healthy cells (negative control) and SH-SY5Y neuroblastoma cells treated with rotenone (positive control), respectively. Results were obtained by the ratio between the red/green fluorescence of the JC-1 dye. The plotted values represent the mean ± SEM of two independent experiments. Statistical analysis was performed by comparing the different treatments with respect to the negative control. (*) corresponds to statistically significant difference with a *p*-value in the range of 0.01 ≤ *p*-value ≤ 0.05, while (**) corresponds to statistically significant difference with a *p*-value in the range of 0.001 ≤ *p*-value < 0.01. The horizontal dotted line represents the membrane potential of the neuroblastoma cells treated with rotenone (* *p*-value ≤ 0.05).

To further gain insights into the neuroprotective role of SC derivatives, we assayed the mitochondrial membrane potential using JC-1 dye. The potential membrane mitochondrial determination allowed us to measure the mitochondrial dysfunction and to understand if the neuroprotective effect of SC derivatives was mediated by biological events on mitochondria. Red fluorescence exhibited by aggregated JC-1 dye indicates healthy mitochondria, while the green fluorescence (monomeric form) is normally observed during apoptotic cell death, due to depolarization processes. Undamaged mitochondria are indicated by an increase in the red/green (R/G) fluorescence intensity ratio [30]. Figure 3A,B presents the results obtained from confocal imaging, where recovered mitochondrial membrane potential can be observed for all SC derivatives treatments assayed. As expected, rotenone-injured cells (positive control) exhibited a significant mitochondrial depolarization with respect to the negative control (healthy cells). Cells treated with SC juice, SC syrup, and Agrosavia NCS exhibited a significant decrease in R/G ratio with respect to the negative control. However, this ratio is significantly higher than that obtained for the positive control. These results provide further evidence of the notion that SC derivatives can reverse the oxidative stress generated by rotenone [31]. Figure 3C shows the fluorescence intensity ratio of Red aggregates/Green monomers. For all concentrations assayed, the SC derivatives showed a ratio above that of the positive control (i.e., around 0.5) and remained around and even above that of the negative one (i.e., around 2) for concentrations up to 250 μg/mL. These results suggest protective effects against mitochondrial membrane potential loss and oxidative stress. A slight reversion in the protective effect was observed for concentrations above 250 μg/mL for the SC juice and SC syrup, which indicate that excess of the compounds might be inhibitory. Further experiments will be needed to elucidate the specific mechanisms that are involved in this phenomenon.

The relatively short incubation time of injured cells with the SC derivatives allowed us to explore their possible protective effect as mitochondria depolarize and early apoptotic processes are triggered [31,32]. Such processes are responsible for the generation of reactive oxygen species (ROS) and the subsequent release of pro-apoptotic molecules [33,34].

**Figure 4:**
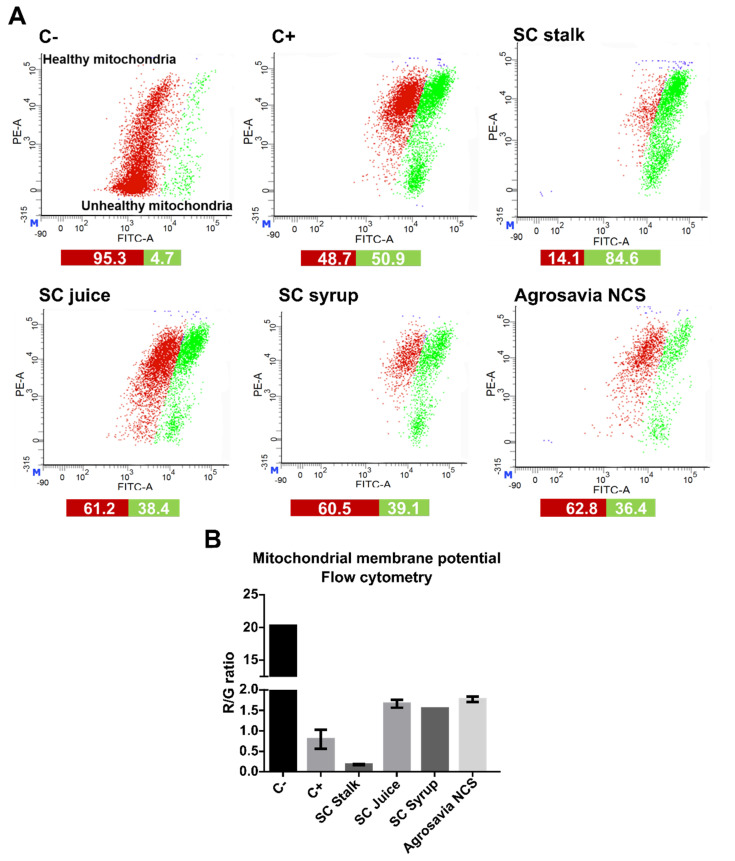
Analysis of mitochondrial membrane potential by flow cytometry on the PD-induced model (SH-SY5Y neuroblastoma cells induced with rotenone) exposed to SC derivatives. Negative control (C-): cells without rotenone treatment. Positive control (C+): rotenone. (**A**) Flow cytometry histograms of PD-induced model labeled with the JC-1 dye after treatment with SC derivatives. (**B**) Quantitative red/green ratio of PD-induced model recovery upon exposure to the SC derivatives (1.59 for SC juice, 1.55 for SC syrup, and 1.72 for Agrosavia NCS), except for SC stalk (0.17).

Further evidence of the neuroprotective effect of SC derivatives was collected from the mitochondrial membrane potential analysis conducted via flow cytometry (Figure 4). According to our results, there was a significant difference between the treatments with all SC derivatives and the injured cells. Surprisingly, the cells treated with the SC stalk showed an elevated proportion of mitochondrial membrane depolarization. These results are not consistent with our observations via confocal microscopy, according to which there was a large R/G ratio associated with a high mitochondrial membrane potential recovery.

### 3.6. Enzymatic Activity of Monoaminoxidase-B (MAO-B) in the PD-Induced Model Exposed to SC Derivatives

**Figure 5:**
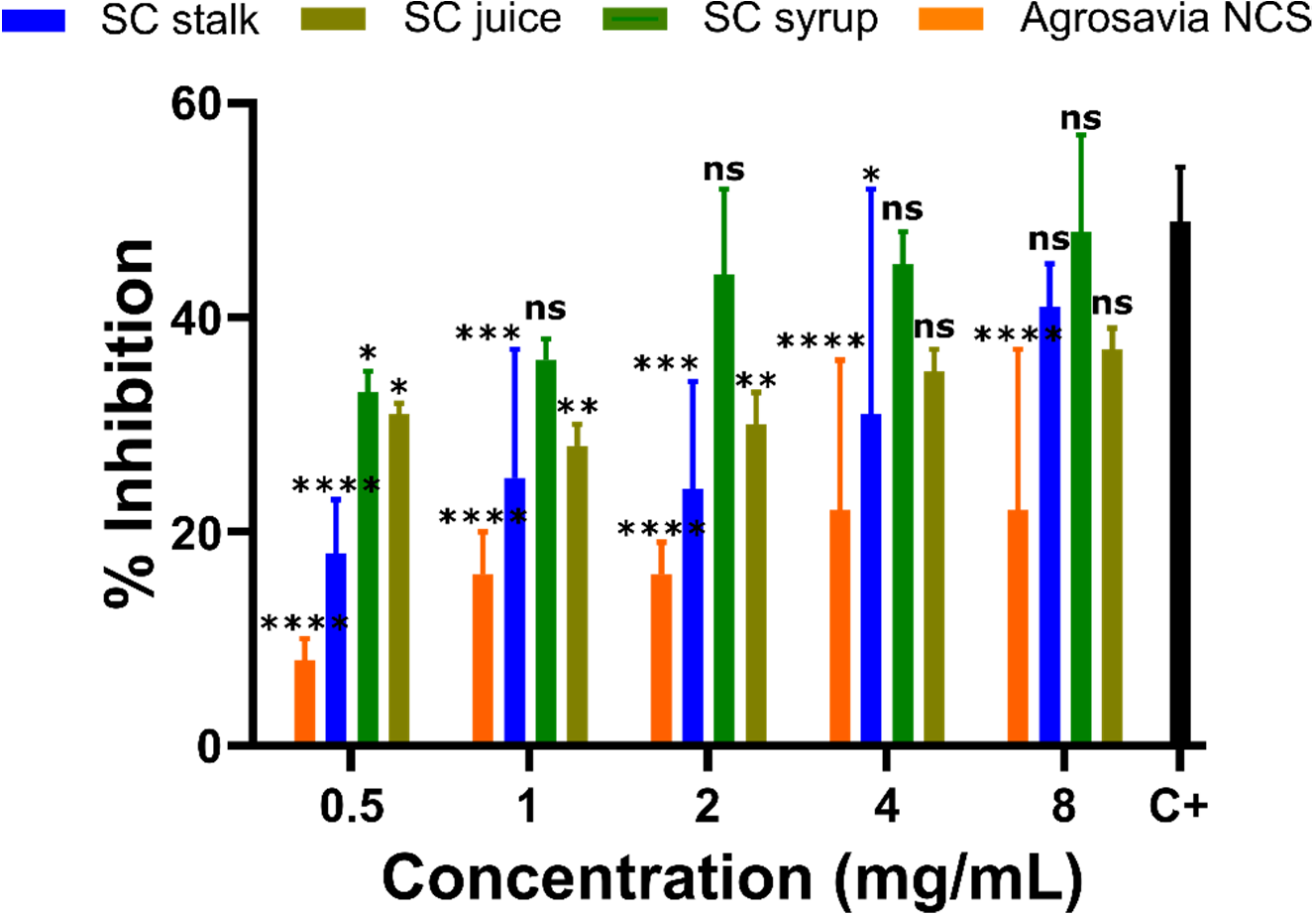
Effect of SC derivatives on MAO-B activity in the PD-induced model (SH-SY5Y neuroblastoma cells). Percentage of MAO-B inhibition relative to the controls (Positive control (C+): rotenone-treated neuroblastoma cells with MAO-B inhibitor pargyline and negative control (C-): rotenone-treated neuroblastoma cells with the reaction buffer (inhibition of 0%)). The values in each column represent the mean ± standard error of the mean (SEM) of three independent experiments. Statistical analysis was performed by comparing the different treatments to the positive control. (*) corresponds to statistically significant difference with a *p*-value in the range of 0.01 ≤ *p*-value ≤ 0.05, (**) to *p-*value in the range of 0.001 ≤ *p*-value < 0.01, (***) to *p*-value in the range of 0.0001 ≤ *p*-value ≤ 0.001, and finally (****) to *p*-value < 0.0001. ns is for treatments where no statistically significant differences were found.

Due to the positive recovery of mitochondrial function found from the mitochondrial membrane potential results shown above, we decided to also measure the activity of the mitochondrial enzyme monoaminoxidase B (MAO-B). This enzyme was selected as its high expression levels have been linked to Parkinson’s disease [35,36]. Our results confirmed that cells treated with rotenone showed high activity of MAO-B, thereby corroborating the expected mitochondrial damage. Overall, Figure 5 shows that the inhibitory impact on MAO-B increases as the concentrations of the SC derivatives increase. Concentrations below 0.5 mg/mL of SC derivatives failed to show inhibition of MAO activity. Moreover, when compared with the pargyline positive inhibition control, Agrosavia NCS showed the lowest inhibitory potential of all SC derivatives, while the SC Syrup showed the largest.

### 3.7. Apoptosis and Cell Cycle Arrest in the PD-Induced Model Exposed to SC Derivatives

**Figure 6:**
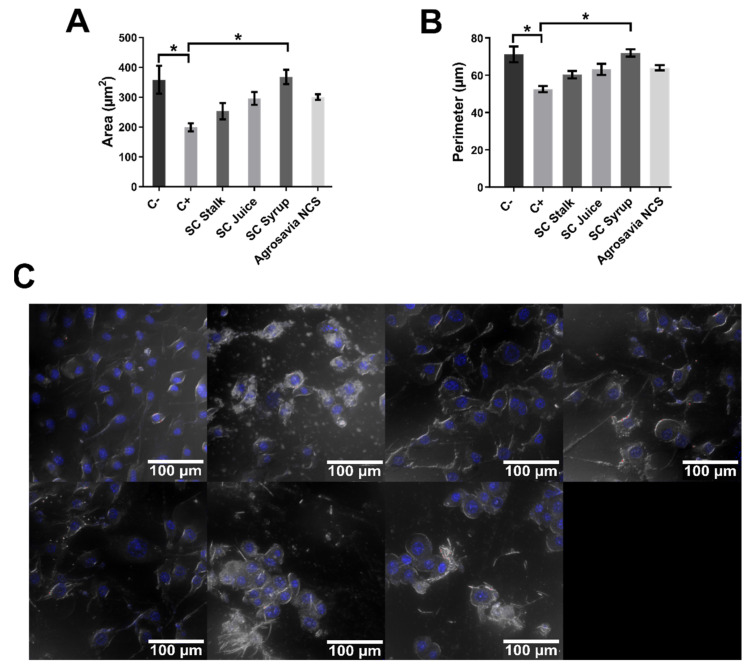
Analysis of morphological changes in PD-induced model nuclei stained with Hoechst dye. Negative control (C-): cells without rotenone treatment. Positive control (C+): rotenone. Quantitative analysis of area and perimeter of cell nuclei was performed over the images presented in Figure 3A. (**A**) Area of cell nuclei exposed to different treatments. The smallest area was obtained for the nuclei of cells exposed to rotenone (198.963 ± 13.845 µm^2^). The largest area was obtained for the control treatment (358.243 ± 46.879 µm^2^). A significant decrease in the nuclear area of the rotenone treatment is observed compared to the control and the SC syrup treatment. (**B**) Perimeter of cell nuclei exposed to different treatments. The smallest perimeter was obtained for the nuclei of cells exposed to rotenone (52.525 ± 1.629 µm). The largest perimeter was obtained for the control treatment (71.202 ± 4.249 µm). A significant decrease in the nuclear perimeter of the rotenone treatment is observed compared to the control and the SC syrup treatment. (**C**) SH-SY5Y neuroblastoma cells treated with rotenone 150 μM for 24 h and exposed to Hoechst dye. Images show that rotenone induced nuclear fragmentation. All the images correspond to the analysis of SH-SY5Y cells after exposure to rotenone (24 h) in the absence of treatments with SC derivatives. Two-way ANOVA test was used to assess statistical significances * (*p*-value ≤ 0.05).

Apoptotic processes were monitored by morphological changes in the nuclei to establish early or late entrance into apoptosis. This was explored with the aid of Hoechst dye, which allows the analysis of morphological changes associated with the beginning of late apoptotic processes [37,38], represented by changes in parameters such as nuclear area and perimeter [39,40]. According to literature data, the nuclear area of SH-SY5Y neuroblastoma cells normally varies in a range of 250–350 µm^2^ [36]. Figure 6A shows that rotenone led to a decrease in the nuclear area with respect to healthy cells, which confirms the beginning of the apoptotic process induced by the oxidative stress [32,37,38,39]. In contrast, the PD-induced model exposed to SC derivatives (SC stalk, juice, and syrup) showed a significant difference in the nuclear area [39,41] when compared with just rotenone exposure. The SC syrup treatment led to a significantly higher nuclear area with respect to injured cells, which provides further evidence for the protective effect of this derivative [32,39]. This notion was supported by the calculated nuclear perimeters, where a similar trend was found (Figure 6B). In addition, Figure 6C shows confocal images of SH-SY5Y neuroblastoma cells 24 h after treatment with rotenone (150 μM).

**Figure 7:**
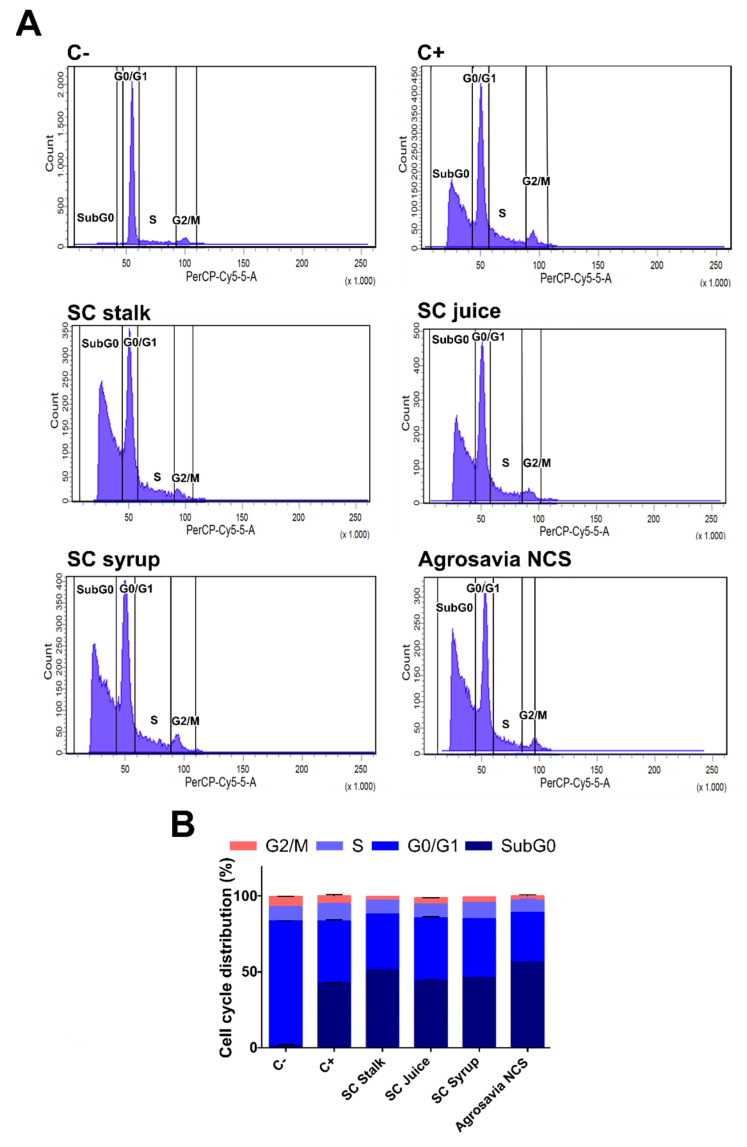
Effect of SC derivatives on cell cycle distribution. Negative control (C-): cells in the absence of rotenone treatment. Positive control (C+): cells in the presence of rotenone. (**A**) Representative flow histograms of the cell cycle distribution in neuroblastoma cells treated with rotenone for a prolonged incubation time. (**B**) Cell cycle distribution percentage in rotenone-treated neuroblastoma cells for a prolonged incubation time.

Finally, we also evaluated the neuroprotective effect of SC derivatives by looking at the cell cycle stage in the PD-induced model cells after exposure to the SC derivatives (Figure 7A,B). The PD-induced model cells were stained with propidium iodide followed by analysis via flow cytometry [29]. The obtained data is presented as the fraction of cells in each phase of the cell cycle i.e., Sub G0, G0–G1, S, and G2/M phases. The sub-G0 phase represents the apoptotic cells, while the S and M represent the cells that underwent mitosis. In the uninduced SH-SY5Y neuroblastoma, one major peak was clearly observed at the G0–G1 phase, which represented 82.6% of the cells. A total of 6.7% of the cells were in the G2/M phase, while 9.0% were in the S phase. Only 1.1% of the cells were considered to be apoptotic cells in the Sub G0 phase. In contrast, prolonged exposure of neuroblastoma cells to rotenone (PD-induced model) led to a decrease in the percentage of cells in the G0-G1 phase to 41.2%. At the same time, the percentage of cells in the sub G0 (apoptotic) increased to 41.2%. Rotenone treatment also reduced the percentage of mitotic cells (those in the G2/M phase) to as low as 5.3%. Our results also revealed that the percentage of apoptotic cells was not significantly reduced after exposure to SC derivatives. Even though based on the cell cycle analysis, no recovery of apoptotic cells after prolonged exposure to rotenone was detected; the damage after short exposure times (i.e., initial stages of oxidative stress) were largely reverted by the SC derivatives.

## 4. Discussion

Here, we report on the presence of twelve (12) different bioactive compounds, including five polyphenolic acids, namely, p-hydroxybenzoic acid, vanillic acid, p-coumaric acid, feluric acid, and rosmarinic acid. Additionally, we identified four flavonoids: luteolin, kaempferol, naringenin, and apigenin and three anthocyanins: 3-rutinoside cyanidin, cyaniding, and pelargonidine in all the SC derivatives evaluated. Recent reports have suggested that these bioactive molecules are capable of neutralizing free radicals, which are generally produced by redox reactions under normal physiochemical conditions or pathological states [2,13,26,42,43,44]. As a result, NCS and other natural products might be interesting not only as dietary supplements, but also as nutraceuticals with the potential of increasing cell survival and even preventing the development of various conditions such as neurodegenerative diseases and cancer [12,45,46]. Accordingly, we decided to evaluate the antioxidant activity of NCS, and some of its intermediary derivatives.

The SC derivatives show radical scavenging activity (as determined by two separate assays), which is most likely due to the presence of phenolic acids and flavonoids (Table 2) [47,48,49]. The difference between the IC_50_ obtained by the two separate assays, i.e., ABTS and DPHH, is most likely due to the higher sensitivity of the ABTS-based assay [50]. A notable observation is the superior antioxidant capacity of the SC syrup in comparison with the other derivatives that could be related with the highest concentration of feluric acid. Although, according to the Folin–Ciocalteu method, the pulverized sugarcane (SC stalk) shows high levels of total phenolic compounds, no correlation was found with the scavenging activity. This is most likely due to the presence of additional oxidizable compounds such as cellulose and vegetal proteins, which might not necessarily exhibit radical scavenging activity [51,52]. The antioxidant activities found for the SC derivatives encouraged us to evaluate their neuroprotective effects. This was accomplished by measuring cytotoxicity, mitochondrial membrane potential, monoaminoxidase B activity, and apoptosis of a PD-induced model (i.e., neuroblastoma cells treated with rotenone) in the presence of the SC derivatives. Interestingly, after exposure to the SC derivatives, cell viability was maintained above about 95% (Figure 1). Additionally, in the PD-induced model, the presence of these compounds reduced cytotoxicity by an average of about 50% (as measured by both MTT and LDH assays) (Figure 2). Figure 2 also confirms that the SC derivatives appear to reverse the mitochondrial complex I (disruption in mitochondrial energy production) damage induced by rotenone. In this regard, similar results were reported by Genovese et al., who demonstrated a potent inhibitory effect toward lipoperoxidation of rat brain homogenates by phenolic extracts obtained from sugarcane juice [48]. Further evidence of the mitochondrial damage reversal was provided by an increase in the mitochondrial membrane potential (as measured by confocal microscopy images of the JC-1 probe) for the PD-induced model cells treated with SC derivatives (Figure 3). These potential benefits to mitochondrial function are appealing for the development of new treatments of brain injury and neurodegeneration [20,53]. Additional evidence of such recovery was provided by flow cytometry analyses of JC-1 labeled cells. Except for raw sugarcane (SC stalk), which showed the highest recovery in the confocal experiments, the flow cytometry analyses corroborated mitochondrial membrane potential recovery (Figure 4). This surprising result might be explained by the fact that, in confocal microscopy, only the fluorescence emitted by mitochondria is taken into account [39] while, in flow cytometry, this fluorescence is altered by changes in morphology of the whole cell [34]. These morphological changes are generated by apoptotic processes and are generally observed in times shorter than those required to observe membrane potential changes [39]. This strongly suggests that cells exposed to sugarcane may have entered late apoptosis processes [39]. These results are also supported by the high percentage of cells in Sub G0 phase after treatment with sugarcane (Figure 7A,B). Intracellular inhibition of MAO-B was increased when the PD-induced model cells were treated with high concentrations of SC derivatives.

Morphological changes in nuclei were also reversed by the presence of SC derivatives, evidenced by an increase in the nuclear region when compared with injured cells (Figure 6). The results also support the idea that treatment with SC derivatives may directly or indirectly reduce the incidence of apoptosis caused by short time exposure to rotenone (Figure 7). These properties are of the utmost importance for any therapeutic strategy intended to slow down or arrest the progression of neuronal loss.

However, the mechanism of neuroprotection conferred by SC derivatives remains unknown. A plausible explanation for these observations is that sugarcane products are likely to enclose a high concentration of molecules belonging to the flavonoid family and to high bioactive phenolic acids such as feluric acid [8,43,47,54]. Flavonoids exhibit anti-inflammatory, antitumor, antiviral, and antiallergic activities, and these have been speculated to be heavily dependent on their intrinsic antioxidative and chelating properties [26,55,56], as well as on their role in the modulation of intracellular signals responsible for cellular survival. Additionally, the results clearly exhibited syrup as the best candidate for future development of nutraceuticals, even over the performance exposed by the final product NCS. This can be explained by the high temperature (over 117 °C) implemented to concentrate the product from syrup to NCS, which can lead to degradation processes of bioactive compounds. Moreover, the high activity of syrup can be explained by the higher concentration of feluric acid, which is a phenolic compound that has been reported to be involved in protection against neurogenerative diseases such as Parkinson’s [57] and Alzheimer’s [58]. In this regard, this compound has been thought to act by different mechanisms, including oxidative stress control by ROS scavenging and modulation of the expression of different molecules such as the intercellular adhesion molecule-1 (ICAM-1), proinflammatory cytokines IL-1β, IL-6, and TNF-αd, the inducible nitric oxide synthase (iNOS), and cyclo-oxygenase-2 (COX-2) [57,59,60]. This supports the hypothesis that controlling elevated temperatures in the production procedures is paramount in the preservation of these bioactive compounds. Therefore, this highlights the relevance of our work in which new technologies are introduced to improve the traditional production methods. Similarly, the results also demonstrate that by modifying the processing conditions to manufacture NCS, it is possible to obtain a more consistent dietary product with high nutraceutical characteristics. However, we are certain that further in vitro studies are required to irrefutably prove the antioxidant and neuroprotective activities of SC derivatives. These include more accurate and detailed extraction methods, such as supercritical fluid extraction to preserve higher amounts of polyphenolic compounds present in the different SC derivatives, Maillard reaction products quantification assay to elucidate the contribution of these compounds on the high bioactivity of SC syrup and NCS, and intracellular ROS measurements, as well as functional evaluation of bioactivity in more accurate cell models, such as primary culture neurons and astrocytes [10,13,61]. Such beneficial attributes are currently in high demand for the preparation of functional food products with proven activity towards mitigating impaired cellular functions. This is also attractive from the commercial viewpoint as consumers are more informed regarding healthy lifestyles and are consequently willing to buy products that potentially bring them long-term health benefits [42].

## 5. Conclusions

An emerging consumer trend is the search for food supplements with potential long-term benefits to health. This has propelled a tendency to use botanicals as a source of such supplements. This is because numerous recent reports have demonstrated that plants are rich in molecules with potential antimutagenic, anticarcinogenic, anti-infection, immunomodulatory, and antioxidant effects. Due to the high abundance of sugarcane crops in Colombia, and the long tradition of medicinal use by local communities, we decided to start a research program to evaluate whether valuable antioxidant molecules in sugarcane can be preserved through a novel process with more controlled heat transfer, such that the obtained products (SC derivatives) might find nutraceutical value. This new processing scheme was introduced to address the thermal decomposition of the contained molecules induced by the traditional high temperature approach that has been in place for decades.

To our knowledge, the present study provides, for the first time, preliminary evidence that SC derivatives obtained by controlled temperature processes showed a remarkable ability to attenuate the oxidative stress caused by rotenone insult in neuroblastoma cells. The protective effects of SC derivatives were most likely due to the presence of antioxidant compounds able to modulate ROS levels by maintaining the mitochondrial membrane potential. Specifically, we identified, by analytical HLPC-MS, five polyphenolic acids, four flavonoids, and three anthocyanins present in all the SC derivatives. Remarkably, SC syrup exhibited a promising bioactivity in terms of low cytotoxicity, mitochondrial membrane potential recovery, notable inhibition of monoaminoxidase B activity, and apoptosis processes reversion. We hypothesize that this superior activity might be related to the presence of ferulic acid, a phenolic compound that has been previously reported to be a potent neuroprotectant. Further studies must therefore be performed to elucidate the underlying mechanism by which this set of compounds exhibit high neuroprotective properties. In addition, it will be crucial to develop novel methodologies to preserve bioactive molecules in the NCS production procedures as well as to analyze the influence of the variety of the SC and the properties of the soil on the total content of these molecules. Finally, our findings suggest that NCS and intermediary compounds (SC stalk, SC juice, and SC syrup) may be useful as neuroprotective agents to potentially prevent neurodegenerative diseases and brain aging.

## Figures and Tables

**Table 1:** SC derivatives polyphenols amount (Folin–Ciocalteu assay) and antioxidant assessment (DPPH and ABTS assays). Ascorbic acid and Trolox were used as reference for the DPPH and ABTS assays, respectively.

Samples	Total Content of Oxidizable Compounds ^a^	DPPH ^b^	ABTS ^c^
SC stalk	2583.8	4.81 ± 0.69	ND
SC Juice	743.9	4.33 ± 0.35	2.49 ± 0.02
SC Syrup	2318.5	4.36 ± 0.37	1.73 ± 0.07
Agrosavia NCS	3623.6	4.28 ± 0.33	2.39 ± 0.074

^a^ ppm caffeic acids; ^b^ radical scavenging activity (IC_50_ mg/mL); ^c^ % ABTS scavenging activity = (1 − (Asample − Acontrol)/Acontrol) × 100.

**Table 2:** Polyphenol composition of SC derivatives extracts determined by analytical HPLC-MS.

Analyte		RT, min	Concentration (mg/kg)							
			SC Stalk	E (%)	SC Juice	E (%)	SC Syrup	E (%)	Agrosavia NCS	E (%)
**Phenolic acids**	*p*-hydroxybenzoic acid	4.2	<0.01	12	<0.01	12	5.1	2	4.4	2
	Vanillic acid	4.3	84.2	2	17.1	2	16.3	2	18.6	2
	*p*-coumaric acid	4.7	8.2	1	<0.01	4	<0.01	4	<0.01	4
	Feluric acid	4.8	1.2	2	2.1	2	2.7	2	2.3	2
	Rosmarinic acid	5	18.4	1	<0.01	9	<0.01	9	<0.01	9
**Flavonoids**	Luteolin	5.6	0.14	1	0.13	1	0.33	1	0.48	1
	Kaempferol	61	1.1	1	0.086	1	0.54	1	0.81	1
	Naringenin	5.9	0.17	9	0.14	9	0.11	10	0.06	10
	Apigenin	6	0.05	12	0.04	12	0.06	12	0.08	12
**Anthocyanins**	3-rutinoside cyanidin	4	1.3	1	3.3	1	3,1	1	1.3	1
	Cyanidin	4.5	0.42	5	1.2	10	3	4	2.2	1
	Pelargonidine	4.8	0.17	9	0.27	1	0.43	1	0.34	1

## Data Availability

Data is contained within the article.
